# Robotic Lateral Pelvic Lymph Node Dissection Could Harvest More Lateral Pelvic Lymph Nodes over Laparoscopic Approach for Mid-to-Low Rectal Cancer: A Multi-Institutional Retrospective Cohort Study

**DOI:** 10.3390/biomedicines11061556

**Published:** 2023-05-27

**Authors:** Jung Hoon Bae, Jumyung Song, Ri Na Yoo, Ji Hoon Kim, Bong-Hyeon Kye, In Kyu Lee, Hyeon-Min Cho, Yoon Suk Lee

**Affiliations:** 1 Division of Colorectal Surgery, Department of Surgery, Seoul St. Mary’s Hospital, College of Medicine, The Catholic University of Korea, Seoul 06591, Republic of Korea; eysi0815@catholic.ac.kr (J.H.B.); cmcgslee@catholic.ac.kr (I.K.L.); 2 Division of Colorectal Surgery, Department of Surgery, Incheon St. Mary’s Hospital, College of Medicine, The Catholic University of Korea, Incheon 21431, Republic of Korea; kjsong2002@naver.com (J.S.); samryong@catholic.ac.kr (J.H.K.); 3 Division of Colorectal Surgery, Department of Surgery, St. Vincent’s Hospital, College of Medicine, The Catholic University of Korea, Suwon 16247, Republic of Korea; ninayoo11@hanmail.net (R.N.Y.); ggbong@catholic.ac.kr (B.-H.K.); hmcho@catholic.ac.kr (H.-M.C.)

**Keywords:** lateral pelvic lymph node dissection, robotic surgery, laparoscopic surgery, rectal cancer

## Abstract

Lateral pelvic lymph node dissection (LPND) is a technically demanding procedure. This study aimed to compare the short-term outcomes of laparoscopic and robotic LPNDs. This multi-institutional retrospective study included 108 consecutive patients who underwent laparoscopic or robotic total mesorectal excision with LPND for locally advanced rectal cancer. There were 74 patients in the laparoscopic and 34 in the robotic groups. The median operation time was longer in the robotic group than in the laparoscopic group (353 vs. 275 min, *p* < 0.001). No patients underwent conversion to open surgery in either group. Pathological LPN metastases were observed in 24 and 8 patients in the laparoscopic and robotic groups, respectively (*p* = 0.347). Although the number of harvested mesorectal lymph nodes was similar (15.5 vs. 15.0, *p* = 0.968), the number of harvested LPNs was higher in the robotic than in the laparoscopic group (7.0 vs. 5.0, *p* = 0.004). Postoperative complications and length of hospital stay were similar (robotic vs. laparoscopic, 35.3% and 7 days vs. 37.8% and 7 days, respectively). Both laparoscopic and robotic LPND are safe and feasible for locally advanced rectal cancers, but robotic LPND showed more harvested lateral lymph node than laparoscopic LPND.

## 1. Introduction

Surgical eradication of the lateral pelvic lymph nodes (LPNs) in rectal cancer has attracted much attention recently. In Japan, total mesorectal excision (TME) combined with lateral pelvic lymph node dissection (LPND) without preoperative chemoradiation (CRT) has been routinely performed in locally advanced mid-to-low rectal cancer [1,2]. On the other hand, preoperative CRT, followed by TME, has been widely accepted as a standard treatment in the same situation in Western countries [3,4]. Two disparate treatment strategies have been performed because of the ambivalence of LPND, which reduces local recurrence and improves survival [5,6] while increasing postoperative morbidity such as sexual or urinary dysfunction [7]. A recent collaborative study on LPNs showed that combining LPND and TME decreased local recurrence in patients with suspected LPN metastasis even after preoperative CRT [6]. Therefore, interest in LPN treatment has been moving toward performing LPND in cases with suspected LPN metastases in both Western and Eastern countries [5,6].

Another concern is the technical aspects of LPND. Despite its potential oncological benefits, LPND is still a procedure many colorectal surgeons are reluctant to perform because of its complexity, difficulty, and potential postoperative morbidity. Rectal cancer surgery challenges have inspired innovations because of the high recurrence rates, high morbidity, and the technical difficulties of operating in the deep and narrow pelvis. Robotic surgery has overcome some of these challenges and has shown promising results [8]. Laparoscopic surgery has been widely accepted as the standard modality for colon cancer [9]. However, the laparoscopic approach for rectal cancer has technical limitations due to the use of straight and fixed instruments. Robotic systems have several advantages over laparoscopic platforms. Robotic systems have endo-wrists, which enable free movement, provide high-quality three-dimensional stereoscopic images and stable traction, and prevent natural hand tremors [10]. Thus, the robotic system could be a better surgical option for LPND than the laparoscopic approach. 

However, few studies have reported the potential advantages of robotic LPND compared to the laparoscopic approach. This study aimed to identify the potential advantages of robotic LPND over laparoscopic LPND by comparing the two approaches. 

## 2. Materials and Methods 

Between January 2015 and December 2021, 108 consecutive patients who underwent laparoscopic or robotic TME with LPND for locally advanced mid-to-low rectal cancer (clinically T3 or N+) and clinically suspected LPN metastasis were included from three study centers: Seoul St. Mary’s Hospital (*n* = 46), Incheon St. Mary’s Hospital (*n* = 34), and St. Vincent’s Hospital (*n* = 28). Inclusion criteria were as follows: 1. Locally advanced rectal cancer (clinically T3 or N+), 2. Mid-to-low rectal cancer (tumor located within 10 cm from anal verge (AV)). 3. Pathologically proven adenocarcinoma, 4. Clinically suspected LPN metastasis, 5. Curative intent surgery (R0 resection) 6. Age < 80 years, 7. Elective surgery. The exclusion criteria were as follows: 1. Early-stage rectal cancer (clinically T1-2 and N-), 2. Upper rectal cancer (tumor located above 10 cm from AV), 3. Unresectable distant metastasis, 4. No magnetic resonance imaging (MRI) before surgery, 5. No suspected LPN metastasis. Data were collected from three hospitals affiliated with the College of Medicine, The Catholic University of Korea. This study was approved by the Institutional Review Board of the Ethics Committee of the College of Medicine, The Catholic University of Korea (XC21RADI0112). The procedures were carried out in accordance with the ethical standards of the committee responsible for human experimentation and with the guidelines of the Helsinki Declaration, 1975, as revised in 1983. All patient records were anonymized and de-identified before the analysis.

Patient data, including demographic and clinicopathological characteristics, were collected from each hospital’s rectal cancer patient registry. All patients included in this study were diagnosed with clinically suspected LPN metastases using pretreatment MRIs. After preoperative CRT, an MRI re-assessment was performed to check the tumor response and change in LPN size. Clinically suspected LPN metastasis was defined as the enlargement of lymph nodes (LNs) greater than 5 mm in short-axis diameter with speculated borders. Mid-to-low rectal cancer was defined as a tumor with an inferior margin within 10 cm from the AV and below the peritoneal reflection, as assessed on MRI (mid, 5.1–10.0 cm; low, 0–5.0 cm) [11]. The numbers of harvested and metastatic LNs in the mesorectum and lateral compartment were pathologically reported. Postoperative complications were recorded within 30 days after surgery using the Clavien–Dindo classification. 

Preoperative CRT was recommended as the initial treatment for all patients with suspected metastatic LPNs. However, it was omitted in some patients who refused preoperative CRT, had distant metastases, or were expected to have low compliance owing to their advanced age or comorbidities. The choice of long-course and short-course radiation was made based on the surgeon’s preference and multidisciplinary team discussion. Surgery was performed 7–8 weeks after completion of any CRT [12]. All surgeries were performed by colorectal surgeons certified in the subspecialty of colorectal surgery by the Korean Surgical Society. Five surgeons who participated in the study belonged to the same university and held monthly meetings to standardize the procedure. The surgery details were the same for both laparoscopic and robotic approaches.

**Figure 1:**
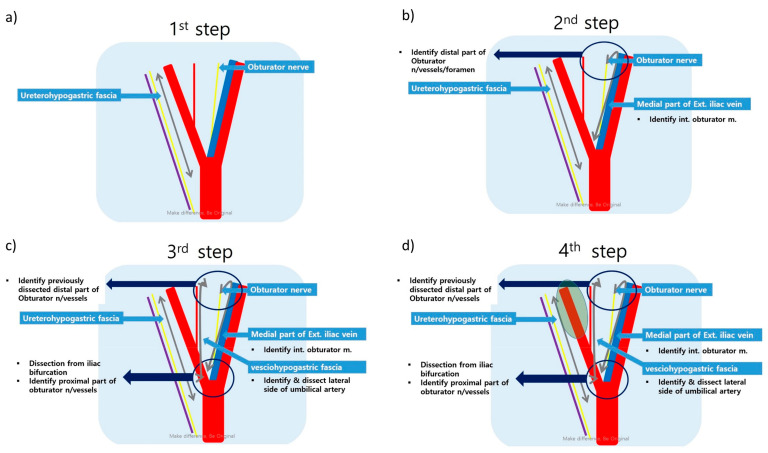
Standardized procedure for right lateral pelvic lymph node dissection: (**a**) Dissection of the uretero-hypogastric fascia; (**b**) Dissection of the obturator space; (**c**) Dissection of the vesico-hypogastric fascia; (**d**) Dissection of the internal iliac vessels.

**Figure 2:**
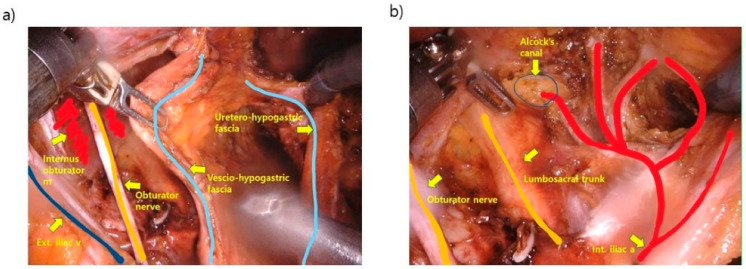
Surgical view after completion of left lateral pelvic lymph node dissection: (**a**) External view of the lateral pelvic area; (**b**) Internal view of the lateral pelvic area.

In the robotic approach, the da Vinci Xi Surgical System (Intuitive Surgical Inc., Sunnyvale, CA, USA) was used with a standardized procedure [13]. Step 1 was the dissection of the uretero-hypogastric fascia, which envelopes the ureter and the hypogastric nerve. Step 2 was the dissection of the medial side of the external iliac vein located at the lateral border of the obturator LN group. Step 3 was the dissection of the vesico-hypogastric fascia at the medial border of the obturator LN group. The final step was the dissection of the internal iliac artery to Alcock’s canal (Figure 1 and Figure 2).

Surveillance was performed every 3–6 months until 2 years postoperatively and then every 6–12 months until 5 years postoperatively. Tumor markers including carcinoembryonic antigen and carbohydrate antigen 19–9, abdominopelvic computed tomography (CT), and chest CT were performed according to the surveillance schedule. Local recurrence was defined as the disease recurrence in the pelvic cavity, including the lateral pelvic area. Local-recurrence-free survival and recurrence-free survival were defined as the interval from the date of surgery until the date of local recurrence and any recurrence detection by radiological or pathological examination or, in case of no recurrence, until the date of last follow-up. The follow-up was completed in April 2023.

Proportions and medians (interquartile ranges (IQR)) are presented for categorical and continuous variables, respectively. Chi-square or Fisher’s exact tests assessed the association between categorical variables and surgical approaches. Continuous variables were compared using independent t-tests. Statistical significance was set at *p* < 0.05. All statistical analyses were performed using SPSS version 25.0 for Windows (IBM Corp., Armonk, NY, USA).

## 3. Results

### 3.1. Baseline Characteristics

A total of 108 patients with rectal cancer who underwent TME with LPND were included in this study. The baseline characteristics are summarized in Table 1. Laparoscopic and robotic LPND was performed in 74 (68.5%) and 34 (31.5%) patients, respectively. The median age was 61 years (IQR 54–68 years), and male and female patients were 71 (65.7%) and 37 (34.3%), respectively. Tumors were located in the mid rectum (5.1 cm to 10.0 cm from the AV) and low rectum (0 cm to 5.0 cm from AV) in 67 (62.0%) and 41 (38.0%) patients, respectively. Preoperative CRT was performed in 84 (77.8%) patients. The two groups had no significant differences between the baseline patient, tumor, or LPN characteristics.

### 3.2. Intraoperative Outcomes

Intraoperative outcomes are shown in Table 2. The ratios of surgical procedures (low anterior resection, intersphincteric resection, and abdominoperineal resection) were not different between the two groups. Bilateral LPND was performed in six (8.1%) and four (11.8%) patients in the laparoscopic and robotic LPND groups, respectively (*p* = 0.722). The median operation time was longer in the robotic group than in the laparoscopic group (353 vs. 275 min, *p* < 0.001), while the estimated blood loss (EBL) was similar *(p* = 0.854). None of the patients in either group required conversion to open surgery.

### 3.3. Pathological Outcomes

Pathological outcomes are shown in Table 3. The pathological T and N stages were similar between the two groups. Pathological LPN metastases were observed in 24 (32.4%) and 8 (23.5%) patients in the laparoscopic and robotic groups, respectively (*p* = 0.347). Although the number of harvested mesorectal LNs was similar, the number of harvested LPNs was higher in the robotic than in the laparoscopic group (7.0 vs. 5.0, *p* = 0.004). The number of metastatic LPNs was similar between the groups. Other pathological factors such as margin status, differentiation, and lymphatic, vascular, and perineural invasion were not different between the groups. 

### 3.4. Postoperative Outcomes

The postoperative outcomes are summarized in Table 4. The median length of the hospital stay after surgery was 7 days (IQR: 6–9 days). There were 40 (37.0%) postoperative complications and 11 (10.2%) major complications, defined as Clavien–Dindo classification ≥3 within 30 days after surgery. There was one case of mortality due to sepsis caused by anastomotic leakage. There were no significant differences between the two groups. The postoperative complications directly related to LPND were lymphocele in four cases and bleeding of the internal iliac artery that required reoperation in the LPND group. The postoperative urinary dysfunction rates in the laparoscopic and robotic LPND groups were 12.2% and 5.9%, respectively. Among them, only one case of lymphocele was observed in the robotic group (*p* = 0.497).

### 3.5. Oncological Outcomes

**Figure 3:**
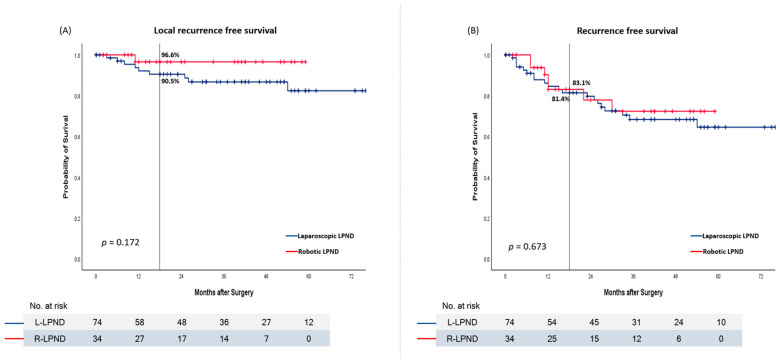
Kaplan–Meier curve in laparoscopic and robotic LPNDs. (**A**) Local-recurrence-free survival. (**B**) Recurrence-free survival.

The local recurrence and recurrence rates were compared between the laparoscopic and robotic LPND groups. No significant differences in local recurrence and recurrence-free survival were observed between the two groups (*p* = 0.172 and 0.673). However, the 18-months local-recurrence-free survival rate tended to be higher in robotic LPND than in laparoscopic LPND. That was 96.6% in robotic group and 90.5% in laparoscopic group. The 18-months recurrence-free-survival rates were 83.1% in robotic LPND and 81.4% in laparoscopic LPND (Figure 3).

## 4. Discussion

This study compared the short-term outcomes between laparoscopic and robotic LPND for locally advanced mid-to-low rectal cancer with suspected LPN metastases. Most perioperative outcomes, such as operative details and pathologic and postoperative outcomes, were not different between the two groups. The number of retrieved LPNs was significantly greater in the robotic group than in the laparoscopic group (7.0 vs. 5.0, *p* = 0.004), while the operating time was significantly longer in the robotic group (353 vs. 275 min, *p* < 0.001).

With the development of minimally invasive surgery, the scope of surgery using robotic systems has gradually expanded. Rectal cancer surgery is one of the most well-established surgeries in terms of the safety and feasibility of the robotic platform [14]. Robotic surgery enables precise dissection in the narrow pelvic cavity compared to the laparoscopic approach. A meta-analysis of randomized controlled trials demonstrated the safety and feasibility of robotic rectal cancer surgery, along with its lower conversion rate to open surgery and longer operating time than the laparoscopic approach [15]. Two other meta-analyses reported similar results. The first showed that the robotic platform was superior to laparoscopy regarding blood loss, open conversion, hospital stay, and postoperative complications, whereas conventional laparoscopy had an advantage regarding operative time [16]. The other also showed that robotic surgery for rectal cancer was superior to laparoscopy in terms of conversion, and the rest of the perioperative outcomes were similar between the two approaches [17]. 

Unlike TME, cumulative evidence of the clinical efficacy of robotic LPND is scanty. Several reports have described the surgical techniques or case series for robotic LPND [13,18,19,20]. However, only one retrospective study compared short-term outcomes between laparoscopic and robotic LPNDs in rectal cancer, in 2018 [21]. Kim et al. demonstrated that robotic TME with LPND is safe and feasible, with favorable surgical outcomes compared to the laparoscopic approach [21]. Although a previous study and ours show similar results in an overall context, there are some differences in details. Although they described no differences in the operating time between laparoscopic and robotic LPNDs, the operating time was longer in the robotic group in the present study. This may be due to differences in the surgeons’ experience participating in each study. In Kim’s study, all the procedures were performed by one highly experienced surgeon. In contrast, five surgeons were included in the present study. Furthermore, several studies that compared laparoscopic and robotic TME have reported a significantly longer operation time in robotic TME; we thought that could explain our longer operation time in the robotic group [22,23]. 

Regarding pathological outcomes, the number of harvested LPNs was significantly higher in the robotic group (7.0 vs. 5.0, *p* = 0.004). Although there is no consensus about the optimal number of harvested lateral pelvic lymph nodes, this finding could be interpreted to indicate that the completeness of the LPND could be better in the robotic approach than in the laparoscopic approach. 

The robotic procedure has the following advantages, in terms of being a surgical technique: it provides fixed third-arm retraction, a magnified three-dimensional view, and endo-wristed instruments. In rectal cancer surgery, both TME and LPND are performed within the narrow pelvic cavity. Therefore, the advantages of robotic surgery in LPND coincide with those of TME in many respects. However, there are some differences between the two technics. TME is dissected along the avascular plane between the mesorectal fascia and the presacral fascia. On the other hand, since LPND only requires dissection of lymphatic tissue, the complex vascular and nerve complexes in the lateral pelvic area should be preserved while skeletalizing. Excessive traction and trembling during lymphatic dissection could cause postoperative urinary dysfunction without direct damage to the major pelvic nerve plexus [7]. During robotic LPND, very stable traction or counter-traction can be performed in the very narrow pelvic side wall, so surgeons can expect less injury to the neural tissue of the lateral pelvic area. Although there was no significant difference in our study, we observed a lower tendency of postoperative urinary dysfunction in the robotic group (12.2% vs. 5.9%). Additionally, fluorescence imaging combined with infrared optics may be helpful to identify metastatic lymph nodes and to reduce the risk of missing hidden pelvic nodes in real time when using the robotic system. 

This study had some limitations. Firstly, because this study was retrospective, inherent and unintentional selection bias could not be dismissed. Secondly, we could not compare the postoperative functional outcomes between the two groups. But to our knowledge, this is the second retrospective study comparing perioperative outcomes between the two approaches to LPND. Thirdly, we reported only a short period of oncological outcomes because the follow-up was too short. However, the 18-months local-recurrence-free survival rate tended to be higher with robotic LPND than with laparoscopic LPND. Regarding pathological outcomes, the number of retrieved LPNs was significantly higher in the robotic surgery group. Based on these findings, we plan to report the long-term oncological outcomes of robotic and laparoscopic LPND in the future.

## 5. Conclusions

In conclusion, the robotic approach is a safe and feasible surgery option for TME with LPND, with more harvested lateral lymph nodes than the laparoscopic approach. A large prospective study would help validate the safety, feasibility, and potential benefits of robotic TME and LPND in locally advanced rectal cancer.

## Figures and Tables

**Table 1:** Baseline characteristics of patients who underwent laparoscopic and robotic total mesorectal excision with lateral pelvic lymph node dissection.

Variables	Total Patients (N = 108)	Laparoscopic LPND (N = 74)	Robotic LPND (N = 34)	*p*-Value
Age (years)	61 (54–68)	63 (54–71)	60 (53–66)	0.129
Sex				0.555
Male	71 (65.7)	50 (67.6)	21 (61.8)	
Female	37 (34.3)	24 (32.4)	13 (38.2)	
BMI (kg/m^2^)				0.111
<25	71 (65.7)	45 (60.8)	26 (46.5)	
≥25	37 (34.3)	29 (39.2)	8 (23.5)	
ASA score				0.233
<3	105 (97.2)	73 (98.6)	32 (94.1)	
≥3	3 (2.8)	1 (1.4)	2 (5.9)	
Tumor level from AV (cm)				0.641
>5	67 (62.0)	47 (63.5)	20 (58.8)	
≤5	41 (38.0)	27 (36.5)	14 (41.2)	
cT stage				0.107
2–3	72 (66.7)	53 (71.6)	19 (55.9)	
4	36 (33.3)	21 (28.4)	15 (44.1)	
cN stage				0.132
1	56 (51.9)	42 (56.8)	14 (41.2)	
2	52 (48.1)	32 (43.2)	20 (58.8)	
cM stage				0.722
0	98 (90.7)	68 (91.9)	30 (88.2)	
1	10 (9.3)	6 (8.1)	4 (11.8)	
LPN location				0.953
Unilateral	95 (88.0)	65 (87.8)	30 (88.2)	
Bilateral	13 (12.0)	9 (12.2)	4 (11.8)	
LPN region				0.104
Internal iliac	66 (61.1)	50 (67.6)	16 (47.1)	
Obturator	32 (29.6)	17 (23.0)	15 (44.1)	
External iliac	8 (7.4)	5 (6.8)	3 (8.8)	
Multiple	2 (1.9)	2 (2.7)	0 (0)	
^a^ Initial LPN size (mm)	10.0 (7.0–13.0)	10.0 (7.5–13.0)	8.0 (6.0–11.0)	0.232
^b^ Initial LPN size (mm)	10.0 (6.4–12.0)	10.0 (7.5–13.0)	7.1 (5.9–11.0)	0.186
^a^ Initial CEA level (ng/mL)	4.3 (9.5–2.3)	4.6 (2.6–11.4)	3.4 (1.8–8.0)	0.808
Preoperative CRT				0.076
No	24 (22.2)	20 (27.0)	4 (11.8)	
Yes	84 (77.8)	54 (73.0)	30 (88.2)	
^b^ Post-CRT LPN size (mm)	6.0 (4.3–8.0)	7.0 (5.0–9.0)	5.0 (3.0–6.2)	0.261
^b^ Post-CRT CEA level (ng/mL)	2.2 (1.4–4.3)	2.3 (1.4–5.0)	2.0 (1.4–2.9)	0.745
Radiotherapy				0.430
Short course	21 (25.0)	12 (22.2)	9 (30.0)	
Long course	63 (75.0)	42 (77.8)	21 (70.0)	
Chemotherapy				0.101
FL	23 (27.4)	18 (33.3)	5 (16.7)	
Capecitabine	61 (72.6)	36 (66.7)	25 (83.3)	

ASA = American Society of Anesthesiologists; AV = anal verge; BMI = body mass index; CEA = carcinoembryonic antigen; CRT = chemoradiotherapy; LPN = lateral pelvic lymph node; LPND = lateral pelvic lymph node dissection. Proportions ( ) are presented for categorical data. Medians and interquartile ranges (IQR) are presented for continuous data. ^a^ median value (IQR) in whole patients. ^b^ median value (IQR) of patients who underwent preoperative CRT.

**Table 2:** Intraoperative outcomes of patients who underwent laparoscopic and robotic total mesorectal excision with lateral pelvic lymph node dissection.

Variables	Total Patients (N = 108)	Laparoscopic LPND (N = 74)	Robotic LPND (N = 34)	*p*-Value
Surgical procedure				0.681
Low anterior resection	66 (61.1)	47 (63.5)	19 (55.9)	
Intersphincteric resection	32 (29.6)	20 (27.0)	12 (35.3)	
Abdominoperineal resection	10 (9.3)	7 (9.5)	3 (8.8)	
LPND direction				0.722
Unilateral	98 (90.7)	68 (91.9)	30 (88.2)	
Bilateral	10 (9.3)	6 (8.1)	4 (11.8)	
Operation time (min)	300 (241–374)	275 (230–347)	353 (285–447)	<0.001
Estimated blood loss (ml)	100 (50–150)	100 (50–120)	100 (50–200)	0.854
Conversion to open surgery	0	0	0	1.000

LPND = lateral pelvic lymph node dissection. Proportions ( ) are presented for categorical data. Medians and interquartile ranges are presented for continuous data.

**Table 3:** Pathological outcomes of the patients who underwent laparoscopic and robotic total mesorectal excision combined with lateral pelvic lymph node dissection.

Variables	Total Patients (N = 108)	Laparoscopic LPND (N = 74)	Robotic LPND (N = 34)	*p*-Value
pT stage				0.999
T0 (CR)	12 (11.1)	8 (10.8)	4 (11.8)	
T1	7 (6.5)	5 (6.8)	2 (5.9)	
T2	29 (26.9)	20 (27.0)	9 (26.5)	
T3	53 (49.1)	36 (48.6)	17 (50.0)	
T4	7 (6.5)	5 (6.8)	2 (5.9)	
pN stage				0.375
N0	55 (50.9)	39 (52.7)	16 (47.1)	
N1	32 (29.6)	19 (25.7)	13 (38.2)	
N2	21 (19.4)	16 (21.6)	5 (14.7)	
LPN metastasis	32 (29.6)	24 (32.4)	8 (23.5)	0.347
No. of harvested LN (mesorectum)	15.0 (12.0–21.5)	15.5 (12.0–23.0)	15.0 (12.0–21.0)	0.968
No. of metastatic LN (mesorectum)	0.0 (0.0–1.0)	0.0 (0.0–2.0)	0.0 (0.0–1.0)	0.733
No. of harvested LN (LPN)	5.5 (3.0–8.0)	5.0 (2.0–7.0)	7.0 (4.0–10.0)	0.004
No. of metastatic LN (LPN)	0.0 (0.0–1.0)	0.0 (0.0–1.0)	0.0 (0.0–0.0)	0.320
Tumor size (cm)	3.0 (1.1–4.5)	3.1 (1.6–5.0)	2.9 (0.5–4.0)	0.131
DRM (cm)	1.8 (1.0–3.0)	2.0 (1.0–3.0)	1.6 (0.5–3.0)	0.321
CRM				1.000
Free (≥1 mm)	100 (92.6)	68 (91.9)	32 (94.1)	
Involvement	8 (7.4)	6 (8.1)	2 (5.9)	
Lymphatic invasion, yes	42 (38.9)	30 (40.5)	12 (35.3)	0.603
Vascular invasion, yes	18 (16.7)	12 (16.2)	6 (17.6)	0.853
Perineural invasion, yes	19 (17.6)	11 (14.9)	8 (23.5)	0.272
Poorly differentiated, yes	10 (9.3)	5 (6.8)	5 (14.7)	0.282

CR = complete response; CRM = circumferential radial margin; DRM = distal resection margin; LN = lymph node; LPND = lateral pelvic lymph node dissection. Proportions ( ) are presented for categorical data. Medians and interquartile ranges are presented for continuous data.

**Table 4:** Postoperative outcomes of the patients who underwent laparoscopic and robotic total mesorectal excision combined with lateral pelvic lymph node dissection.

Variables	Total Patients (N = 108)	Laparoscopic LPND (N = 74)	Robotic LPND (N = 34)	*p*-Value
Postoperative hospital stay (days)	7 (6–9)	7 (6–8)	7 (5–11)	0.932
Postoperative complication within 30 days after surgery (CDC)	40 (37.0)	28 (37.8)	12 (35.3)	0.799
1	3 (2.8)	3 (4.1)	0 (0.0)	
2	26 (24.1)	19 (25.7)	7 (20.6)	
3	6 (5.6)	3 (4.1)	3 (8.8)	
4	4 (3.7)	3 (4.1)	1 (2.9)	
5	1 (0.9)	0 (0.0)	1 (2.9)	
Major complication ≥ CDC 3	11 (10.2)	6 (8.1)	5 (14.7)	0.317
Postoperative complication details				
Anastomosis leakage	13 (12.0)	8 (10.8)	5 (14.7)	0.542
Urinary dysfunction	11 (10.2)	9 (12.2)	2 (5.9)	0.497
Wound complication	7 (6.5)	5 (6.8)	2 (5.9)	1.000
Lymphocele	4 (3.7)	3 (4.1)	1 (2.9)	1.000
Ileus	5 (4.6)	2 (2.7)	3 (8.8)	0.323
Anastomosis bleeding	2 (1.9)	2 (2.7)	0	1.000
Internal iliac a. branch bleeding	1 (0.9)	1 (1.4)	0	1.000
Mortality within 30 days after surgery	1 (0.9)	0	1 (2.9)	1.000

CDC = Clavien–Dindo classification; LPND = lateral pelvic lymph node dissection. Proportions ( ) are presented for categorical data. Medians and interquartile ranges are presented for continuous data.

## Data Availability

The data presented in this study are available on request from the corresponding author. The data are not publicly available due to patients’ privacy concerns.
